# Psychological Adaptive Mechanism Maturity, Age, and Depression Symptoms in Advanced-Stage Cancer Patients

**DOI:** 10.3389/fpsyg.2021.718476

**Published:** 2021-10-26

**Authors:** Thomas Beresford, Patricia U. Teschke, Daniel Hipp, Patrick J. Ronan

**Affiliations:** ^1^School of Medicine, University of Colorado, Aurora, CO, United States; ^2^Rocky Mountain Regional VA Medical Center, VA Eastern Colorado Health Care System, Aurora, CO, United States; ^3^SSM Health Care, St. Louis, MO, United States; ^4^Sanford School of Medicine, University of South Dakota, Vermillion, SD, United States; ^5^VA Medical Center-Sioux Falls, Sioux Falls, SD, United States

**Keywords:** psychological adaptive mechanisms, age, adaptation, cancer, depression

## Abstract

**Background:** Previously, we reported that the maturity of Psychological Adaptive Mechanism (PAM; alternatively, ego defense mechanism) endorsement, but not depression symptom severity, predicted 5-year survival rates in adult cancer patients and that study controlled for age as a significant variable. In this investigation, we hypothesized that greater PAM maturity would correlate significantly with age and with fewer depression symptoms in a larger sample.

**Methods:** In this cross-section study, adult cancer outpatients (*N*=293) completed the Defense Style Questionnaire (DSQ), the Beck Depression Inventory (BDI), and provided additional clinical data. Spearman’s correlation and multiple regression modeling provided statistical tests of the study hypotheses.

**Results:** Contrary to our hypothesis, DSQ PAM maturity endorsement did not correlate significantly with increasing age. Greater PAM maturity ratio on the DSQ (*p*<0.0001) and current antidepressant use (*p*<0.05), however, both provided inverse associations with total BDI symptom frequency (*p*<0.01). Age was inversely associated with BDI mood (*p*<0.0001) and somatic scores (*p*<0.04). Items that worsened BDI symptom frequency included self-reported mood-altering anti-cancer medications and any psychiatric history. Cancer stage, time since diagnosis, and chemotherapy treatment did not correlate with DSQ or BDI scores. Multiple regression analysis found that the correlated items accounted for 17.2% of the variance in mood symptoms and 4.9% in somatic symptoms. Specifically, adaptive maturity and age associated with fewer depression symptoms, while cancer medications affecting mood, and a previous psychiatric history each predicted higher frequency of depression scores.

**Conclusion:** The results suggest that PAM maturity likely predicts fewer depression symptoms while younger age associates with more depression symptoms in this clinical sample. Centrally, acting cancer medications, such as glucocorticoids, and any history of psychiatric disorder correlated with increased depression symptom frequencies. In this cross-section study, antidepressant medications indicated higher frequencies of depressive symptoms, likely reflecting their use in persons previously diagnosed with depression. Further research should target factors that improve PAM maturity as a potential treatment target, especially in younger age groups.

## Introduction

In a previous study, we reported that the Maturity level of Psychological Adaptive Mechanisms (PAMs; alternately, “ego defense mechanisms”) independently predicted survival in a small sample of late-stage cancer patients scoring at the extremes on the Defense Style Questionnaire (DSQ) while extreme scores on the Beck Depression Inventory (BDI) did not (Beresford et al., 2006). In that sample, half of those who endorsed mature adaptive styles survived for 5years, whereas half of those endorsing immature PAMs survived only 18months. The study identified age as a significant confounding variable for which our statistical analysis controlled in arriving at those final survival data. The relationship between PAMs and age in this setting remains poorly understood, however, and to the best of our knowledge, no studies have examined hierarchical PAM maturity effects in relation to age and depression in cancer patients (Di Giuseppe et al., 2018).

In elegant longitudinal studies of non-pathologic samples followed over many decades, Vaillant observed that psychological adaptation matures over time, as primitive adaptive mechanisms evolve “into more mature mechanisms, rather than being abandoned or replaced” (Vaillant et al., 1986). His observations suggested that older age might predict greater maturity score endorsement when assessed in a clinical sample, such as in those suffering from cancer. That is, hypothetically, psychological and temporal maturity go hand in hand.

Posited links between age and depression in cancer patients continue to provide cause for debate. For example, large studies using the Hospital Anxiety and Depression Scale (HADS) reached conflicting results. One found depression to increase with age and another found depression more likely in younger rather than older adults (Carroll et al., 1993; Hinz et al., 2010). Yet another study found no age effect on depression occurrence among cancer patients (Aass et al., 1997). In the present study, we hypothesized that positive associations would occur between increasing age and greater PAM maturity, as well as, between increasing age and fewer depression mood symptoms in this sample of late-stage cancer patients.

## Materials and Methods

### Study Design

This cross-section study analyzed data collected during summer months from 1999 through 2010. Fellowship grants from the National Cancer Institute through the University of Colorado Cancer Center and the Department of Psychiatry of the University of Colorado School of Medicine funded this project. Summer research assistants were trained and certified in institutional requirements with respect to confidentiality, research ethics, and good clinical practices. All research assistants were supervised by the principal investigator (TB) who also supervised data collection as did permanent research staff members. All the research assistants received specific training in the administration of each of the instruments used in this study.

Prior to data collection, the research assistants explained the parameters of the study to potential subjects and obtained informed consent. Subjects who consented to the study then underwent a delirium screening examination and were asked to rate their pain, if present, on a linear-analog scale. Participants not excluded for either delirium or significant pain completed a brief demographic questionnaire. Finally, chart reviews verified items related to participants’ medical history, such as cancer diagnosis and stage.

### Participants

Study participants included cancer outpatients entered consecutively during the respective time periods. All participants were adults who consented to this Institutional Review Board approved study. In order to meet inclusion criteria for this study, participants: (1) presented with a diagnosis of cancer; (2) were free of delirium as assessed by a standard delirium scale (Trzepacz et al., 1988); (3) were either pain free or presented with minimal pain, as described below; and (4) agreed to participate voluntarily. Twenty potential subjects declined participation (*N*=20 of 315); 14 provided no reason, four said the process was too long, and two reported feeling too tired. In accordance with privacy regulations, no further data were collected on those who declined consent.

Participants suffering from pancreatic or neuro-humoral secreting neoplasm, such as carcinoid or pituitary tumors, were excluded because of the known effects of these neoplasms on mood and affect. No patients were excluded due to pain of sufficient magnitude as to be subjectively distracting or greater than the first quartile of a linear-analog scale. Two patients who failed the delirium screen were excluded from the study. In this way, we accrued a total sample of 293 subjects who provided responses to the study instruments.

### Study Measures

Participants provided demographic and medical data, which included type and stage of cancer and time since cancer diagnosis. Diagnosis and staging were verified by medical record review. Staff also documented whether participants had a prior psychiatric history or reported current antidepressant use, and the patient’s own report of any other current anti-cancer medications that affected their mood.

#### Defense Style Questionnaire

The DSQ was first developed by Bond and validated in subsequent studies as a qualitative and quantitative measure of PAMs (Andrews et al., 1993; Perry and Hoglend, 1998; Bond, 2004). It is a 40 item questionnaire that lists behavioral strategies by which individuals adapt to stressful events in their lives. This questionnaire presents 20 different adaptive styles, with two items for each PAM. Subjects are asked to agree or disagree with each statement on a 9-point Likert-type scale and a mean score for each mechanism is obtained by averaging the scores of the two items corresponding to each adaptive style.

Based in part on Vaillant’s empirical work (Vaillant, 1971, 1985; Soldz and Vaillant, 1998), Bond and colleagues assigned DSQ scores into three groups of psychological adaptive styles: immature, neurotic, and mature (Andrews et al., 1993). Stratifying the scores in this manner allows calculation of category means for each of the three groups for each participant.

#### Beck Depression Inventory

The BDI was developed by Beck and colleagues and validated by several studies as a tool for measuring frequency and severity of depressive symptoms (Beck et al., 1961; Beck and Steer, 1984; Enns et al., 1998; Richter et al., 1998; Aben et al., 2002). The BDI presents questions on specific depressive symptoms and asks respondents to rate their occurrence on a scale ranging from “rarely” to “often.” Each item is scored, yielding a summed total BDI score. In this version of the BDI, mood symptoms (items 1–14) can be separated from somatic symptoms (items 15–21) and analyzed separately.

### Statistical Analysis

Calculation of the mean scores for each of the three DSQ domains (immature, neurotic, and mature) for each participant allowed analysis of the relationships among PAM maturity, age, and depression symptoms. The DSQ calculation added up the points corresponding to each category and divided the sum by the number of adaptive mechanisms corresponding to that domain. For example, in order to calculate the mature mean score, the scores corresponding to sublimation, humor, anticipation, and suppression on the DSQ were added together and then divided by 4. Next, we calculated an adaptive maturity ratio score for each subject by dividing each mean “Mature” DSQ score by the respective mean “Immature” DSQ score.

To facilitate descriptive analyses of time since diagnosis, those data were assigned codes as the follows: 1 for greater than 6 months from date of diagnosis to the date study instruments were completed, 2 for less than 6 months but greater than 1 month, 3 for less than 1 month but greater than 2weeks, and 4 for less than 2weeks.

Calculated Spearman’s correlation determined the presence and statistical power of correlations between variables. This was followed by a multiple regression analysis to characterize predicted relationships.

## Results

### Descriptive Statistics

This study included 293 cancer outpatients (Table 1), ranging in age from 22 to 87years (*M*=56.5, *SD*=11.6). Participants included 156 females (53.2%) and 137 males (46.8%). By cancer stage, 20 participants were classified as Stage I (6.8%), 25 as Stage II (8.5%), 65 as Stage III (22.2%), and 174 as Stage IV (59.4%); nine participants (3.1%) had neoplasms for which stage was undetermined or unavailable. Data were collected on cancer diagnosis for each participant; however, frequency of each type was not evaluated, as the wide variety of cancer diagnoses made direct analysis by type unfeasible. Examples of cancer diagnoses in this sample included the following: breast, ovarian, prostate, testicular, colon, rectal, gastric, esophageal, oral, lung, brain, skin, and hematological malignancies.

**Table 1:** Age, gender, and cancer stage distribution of study sample.

	n	%
Age		
< 40	25	8.53
40–59	138	47.10
60–79	121	41.30
>79	6	2.05
Data Missing	3	1.02
Gender		
Females	156	53.24
Males	137	46.76
Stage		
I	20	6.83
II	25	8.53
III	65	22.18
IV	174	59.39
Data missing or N/A	9	3.07
Total	**293**	**100**

### Main Hypotheses

Adaptive maturity and age: No direct correlation appeared between age and adaptive maturity. Cancer stage, time since diagnosis, and current chemotherapy did not reach correlation significance with either PAM maturity or total BDI scores; therefore, these variables were not included in follow-up analyses. Age was correlated with gender, which reflects the study cohort in which female participants were younger than males.

Age and Depressive Symptoms: A Spearman’s rank correlation was performed to determine the presence and extent of correlations between variables (Table 2). Age significantly, and inversely, correlated with total BDI scores (*r*=−0.23, *p*<0.0001), as well as BDI subscale mood symptom scores (*r*=−0.26, *p*<0.0001) and somatic symptom scores (*r*=−0.12, *p*<0.04). Factors associated with BDI-mood symptom scores included as: Self-reported current anti-neoplastic medications that affect mood (*r*=0.21, *p*<0.001), any psychiatric history (*r*=0.15, *p*<0.01), and adaptive maturity ratio (*r*=−0.24, *p*<0.0001).

**Table 2:** Intercorrelations among predictor and dependent variables (*N*=293).

	1	2	3	4	5	6	7	8	9	10	11	12
1												
2	−0.040											
3	−0.228^**^	−0.271^**^										
4	−0.260^**^	−0.241^**^	0.860^**^									
5	−0.123^*^	−0.234^**^	0.859^**^	0.498^**^								
6	−0.099	0.026	0.201^**^	0.214^**^	0.131^*^							
7	−0.017	−0.033	0.138^*^	0.154^**^	0.057	0.290^**^						
8	−0.038	0.001	0.083	0.136^*^	−0.001	0.352^**^	0.468^**^					
9	0.139^*^	−0.095	−0.075	−0.083	−0.034	−0.082	−0.113	−0.181^**^				
10	0.001	0.049	0.053	0.028	0.073	0.037	−0.029	0.033	0.075			
11	−0.003	0.019	0.049	0.012	0.081	0.062	0.065	0.040	−0.012	−0.071		
12	−0.141^*^	0.020	0.044	0.059	0.024	0.044	−0.010	−0.046	−0.024	0.044	0.049	

1, age; 2, mature/immature ratio; 3, BDI total scores; 4, BDI-mood scores; 5, BDI-somatic scores; 6, self-reported cancer medications affecting mood; 7, psychiatric history; 8, current antidepressant use; 9, gender; 10, cancer stage; 11, time since diagnosis; and 12, current chemotherapy.

* Correlation is significant at the 0.05 level (two tailed).

** Correlation is significant at the 0.01 level (two tailed).

Counterintuitively, current antidepressant use (*r*=0.14, *p*<0.02) and depression symptoms were positively and significantly associated, meaning that antidepressant use indicated more, rather than fewer, BDI-mood symptoms.

### Exploratory Analyses

Associations with BDI-Mood Symptom Variables: To explore these data further, we hypothesized that (1) greater adaptive maturity each would predict lower frequencies of depressive mood symptoms and (2) that any psychiatric history and cancer medications reported as affecting mood, each, would predict more frequent mood and somatic depressive symptoms.

A multiple regression analysis tested these hypotheses (Table 3). Results of the analysis indicated that the overall model was significant, ∆*F* (4,283)=15.87, *p*<0.001, and accounted for 17.2% of the overall variance in BDI-mood subscale scores (Adj. *R*^2^=0.172). More specifically, age (*t*=4.76, *p*<0.001) and PAM maturity (*t*=4.71, *p*<0.001) inversely correlated with the BDI-mood ratings. Cancer medications affecting mood (*t*=2.06, *p*<0.05) and a previous psychiatric history (*t*=2.71, *p*<0.01) each indicated worsening BDI-mood subscale scores. These findings confirmed their respective hypotheses and suggested that older cancer patients and those who employ more mature coping styles are less likely to endorse depressive mood symptoms than younger patients, or those with less mature coping mechanisms.

**Table 3:** Multiple regression for age, mature/immature ratio, medications affecting mood, and prior psychiatric history^a^, and BDI cognitive scores^b^.

Variables	Adj. *R*^2^	Δ*R*^2^	*F*	*B*	*SE*	*β*	sig.
(Constant)	0.172	0.183	15.87	10.615	1.131		0.000
Age				−0.079	0.017	−0.257	0.000
Mature/Immature ratio				−1.329	0.282	−0.254	0.000
Medications affecting mood				0.881	0.428	0.121	0.041
Psychiatric Hx				1.654	0.610	0.159	0.007

a Predictor variables: age, mature/immature ratio, medications affecting mood, and psychiatric history.

b Dependent variable: BDI cognitive scores.

Associations With BDI-Somatic Symptom Variables: We further hypothesized that adaptive maturity, age, medications affecting mood, and psychiatric history would significantly predict the BDI-somatic depressive symptoms. A second multiple regression analysis tested this (Table 4). Results indicated that the overall model was significant, [∆*F* (4,283)=4.72, *p*=0.001], accounting for 4.9% of the overall variance in BDI-somatic symptoms (Adj. *R*^2^=0.049). However, only adaptive maturity (*t*=3.43, *p*=0.001) and cancer medications affecting mood (*t*=1.96, *p*<0.05) were significantly predictive of BDI-somatic symptoms. Age (*t*=1.78, *p*=0.077) and previous psychiatric history (*t*=0.057, *p*=0.95) did not predict somatic scores.

**Table 4:** Multiple regression for age, mature/immature ratio, medications affecting mood, and prior psychiatric history^a^, and BDI-somatic scores^b^.

Variables	Adj. *R*^2^	*ΔR* ^2^	*F*	*B*	*SE*	*β*	sig.
(Constant)	0.049	0.062	4.72	8.920	1.085		0.000
Age				−0.028	0.016	−0.103	0.077
Mature/Immature ratio				−0.930	0.271	−0.198	0.001
Medications affecting mood				0.803	0.411	0.123	0.052
Psychiatric Hx				−0.034	0.586	−0.004	0.954

a Predictor variables: age, mature/immature ratio, medications affecting mood, and psychiatric history.

b Dependent variable: BDI cognitive scores.

## Discussion

Results revealed that both lower PAM maturity and younger age predicted depression mood symptoms in cancer patients, supporting our hypothesis that increasing age and PAM maturity are independently associated with fewer mood symptoms. However, contrary to our hypothesis, age alone did not directly associate with PAM maturity scores. To our knowledge, this is the first study that has investigated lack of an association between age and PAM maturity specifically in cancer patients.

### Limitations

This study’s limitations include its seasonal funding support. Stable funding for a continuous period of time would allow for a more systematic sampling and recruitment of a larger participant population and inclusion of one or more appropriate comparison groups. Furthermore, the study instruments used in this investigation provided ease of use as a best compromise in assessing complex target symptoms and behaviors. The DSQ remains a rudimentary tool for assessing PAMs (Perry and Hoglend, 1998). The senior author has developed a clinical recognition algorithm to address this imprecision, a method that itself requires validity and reliability testing (Beresford, 2012, 2014).

The BDI scores indicate depressive symptom frequency and severity, but the BDI does not diagnose clinical depressive disorders. The BDI version used in this study was the revised version 1, chosen in our view for a potentially greater relevance to a cancer patient sample. Subsequent revisions to the BDI replaced weight loss, body image changes, and somatic preoccupation with concentration difficulty, worthlessness, and loss of energy items, yielding the BDI II version. Studies in non-cancer samples have found small differences in mean ratings for certain symptoms of depression and overall slightly higher mean scores for BDI II compared to its earlier version (Beck and Steer, 1984; Beck et al., 1996).

Like most other studies in this field, the present study offers only a cross-section assessment of its target variable. In respect to age alone, cross-section studies present clear limitations in establishing the variables associated with age that can inform the care of cancer patients. One study reported that younger cancer patients are more likely to endorse depression mood symptoms, a finding consistent with several studies, but contrary to others (Given et al., 1994; Kurtz et al., 1994; Smith et al., 2002; Mystakidou et al., 2005; Wong-Kim and Bloom, 2005; Agarwal et al., 2010; Lo et al., 2010). A large study of 1,529 cancer inpatients in cross-section and 2037 controls found that younger adults with cancer scored significantly higher in depression compared to the age-matched control group (Hinz et al., 2010). But in that study, the difference disappeared in older age groups, suggesting possibly higher baseline depression rates in older patients. Conversely, a number of studies report a positive association between age and depression (Carroll et al., 1993; Smith et al., 2002; Mystakidou et al., 2005), while others reported no age effect (Given et al., 1994; Kurtz et al., 1994; Aass et al., 1997).

Part of the difficulty in assessing depression in cancer patients is symptom overlap. Both advanced-stage cancers and the side effects of chemotherapy may include anorexia, cachexia, sleeping difficulties, and fatigue, all of which may closely resemble the somatic symptoms of depression. While the distinction may be more apparent in a clinician conducted assessment, we have yet to find standardized questionnaires that can easily distinguish depression-related physical symptoms from cancer-related depression-like symptoms.

### Clinical Context

The BDI has been widely used in studies involving cancer patients (Leigh et al., 1987; Richardson et al., 1990; Chang et al., 2004; Mainio et al., 2005, 2006; Beresford et al., 2006). One advantage is its ability to separate mood and somatic symptoms of depression, allowing interpretation in the context of cancer. For instance, one study that investigated the somatic versus mood subscales in 213 cancer patients versus controls found that the inter-group score differences occurred in the somatic scores (Wedding et al., 2007). Another study compared BDI scores for cancer inpatients with their healthy next of kin and with physically healthy patients hospitalized for suicide attempt (Plumb and Holland, 1977). BDI-somatic symptoms significantly differentiated hospitalized and non-hospitalized subjects, but not cancer inpatients from suicidal patients. The BDI-mood scale did not separate cancer inpatients from next of kin; however, both groups scored significantly lower than the suicide attempt group. While these findings support the prominence of somatic symptoms in depression, they also raise questions about the specificity of the somatic scale in the absence of mood symptoms in the setting of cancer.

The disparate reports on the effect of age on depression among cancer patients may reflect the lack of standardized depression assessment tools for this clinical population, as well as differences in study designs and sample populations. It may, however, also indicate that other unaccounted variables could be playing a role in this association. This evidence taken together with our findings that both mature adaptation and increased age predicted fewer depression mood symptoms in cancer patients could suggest a possible role of PAM maturity often unaddressed clinically.

Vaillant’s longitudinal studies found PAM maturity to increase age (Vaillant, 1971, 1985; Vaillant et al., 1986). Some cross-section studies have reported a negative association between age and endorsement of maladaptive mechanisms (Whitty, 2003). Others have found no age differences in younger and older adults (Tuulio-Henriksson et al., 1997; Segal et al., 2007). Change versus stability of adaptive mechanisms likely requires longitudinal study design rather than cross-section exploration, particularly among young adults (Diehl et al., 1996; Tuulio-Henriksson et al., 1997; Whitty, 2003; Segal et al., 2007; Yu et al., 2008). Since the previous studies mentioned here involved non-clinical populations, it is difficult to predict the comparability of their findings in a clinical sample, particularly in the setting of cancer.

Immature PAMs have been associated with adverse outcomes in Sjogren’s disease (Hyphantis et al., 2011), multiple sclerosis (Hyphantis et al., 2008), inflammatory bowel disease (Hyphantis et al., 2005, 2010), chronic obstructive pulmonary disease (Albuquerque et al., 2011), and diabetes mellitus (Martino et al., 2020). Endorsement of immature mechanisms has also been linked to depression (Akkerman et al., 1992; Bond, 2004; Bronnec et al., 2005; Blaya et al., 2006; Sharma and Sinha, 2010) and anxiety (Bond, 2004; Blaya et al., 2006) in the general population, but there are only limited data in cancer patients. One study of 100 patients with primary liver cancer reported a positive correlation between depressive symptoms and endorsement of immature defense mechanisms, consistent with our present findings (Wan et al., 2003).

Although we could find no other studies investigating the role of age in adaptative maturity and depression in cancer patients, there have been a few reports on the role of coping styles in this setting. A study of 199 early stage lung cancer patients undergoing surgical intervention found an inverse correlation between depressive symptoms and age, and between depressive symptoms and adaptive coping styles (Walker et al., 2006). Another in 80 women with breast cancer also found higher depressive symptom endorsement in younger patients and in those who demonstrated maladaptive coping styles (Compas et al., 1999). While coping styles and adaptive mechanisms are measured differently, these indirect findings further support a role for psychological adjustment mechanisms in respect to age and depression.

### Clinical Application

Statistical models presented above accounted for a total of about one-fifth of the variance in BDI mood and somatic symptoms in this study. Future research should focus on identifying these variables and how they affect younger versus older adult cancer patients’ depression. For everyday clinical use, three of the associated variables bear mention. First, the self-report of cancer therapy medications affecting mood appeared to influence the production of depression symptoms. While this warrants further analysis, our first impression suggests that untreated glucocorticoid effects may contribute a significant clinical share. Second, any psychiatric history appeared to contribute its weight in ways that require further delineation of specific histories and conditions. Last, and most surprising on its face, the presence of an antidepressant medication appeared to increase, rather than lessen, the BDI symptom frequencies. The relationship may not have been causal, however, since more depression symptoms indicate antidepressant intervention clinically and antidepressant effectiveness is outside the scope of this study.

An overall suggestion from this study’s data, therefore, may serve to sharpen the focus of improving depression treatment in cancer patients (1) who show less mature PAMs, (2) who are younger rather than older, (3) who report mood changes on the anti-neoplastic medications, and (4) who report a preexisting psychiatric history. Improved clinical care will require PAM characterization of subgroups of cancer patients for whom specific treatments, such as supportive or other forms of psychotherapy, can result in significant improvement during cancer diagnosis and treatment.

### Conclusion

While age alone does not predict PAM maturity in the setting of cancer, both advanced age and greater PAM maturity, independently, lessen the risk of depression in cancer patients. While age is independent of treatment, PAMs invoke the possibility of active treatment aimed at increasing PAM maturity and lessening the effects both of cancer and the depression related to it. Effecting positive changes in PAM maturity point toward targeted psychotherapy as a potential specific treatment in cancer conditions. Whether achieved through experience or psychological treatment, PAM maturity can lessen the psychological damage of cancer and in so doing potentially prolong survival.

## Data Availability Statement

The raw data supporting the conclusions of this article will be made available by the authors, without undue reservation.

## Ethics Statement

The studies involving human participants were reviewed and approved by the Colorado Multiple Institutional Review Board. The patients/participants provided their written informed consent to participate in this study.

## Author Contributions

TB: principal investigator and author. PT: co-investigator, data manager, and co-author. DH: co-investigator, data analysis, and co-author. PR: editor, co-investigator, reference coordinator, and co-author. All authors contributed to the article and approved the submitted version.

## Funding

Dr.’s Beresford and Ronan receive partial support from Department of Veterans Affairs Merit Review Award, I01BX004712. Dr. Ronan receives support from NIMH 1 R01 MH122954 (P.J.R.), NIH NIGMS U54GM128729, both through the Great Plains Veterans Research Foundation.

## Disclaimer

The views expressed in this article are those of the authors and do not necessarily reflect the position or policy of the Department of Veterans Affairs or the United States government.

## Conflict of Interest

The authors declare that the research was conducted in the absence of any commercial or financial relationships that could be construed as a potential conflict of interest.

## Publisher’s Note

All claims expressed in this article are solely those of the authors and do not necessarily represent those of their affiliated organizations, or those of the publisher, the editors and the reviewers. Any product that may be evaluated in this article, or claim that may be made by its manufacturer, is not guaranteed or endorsed by the publisher.
